# An Artifact-Free Assay for the GSH/GSSG Ratio Adapted for Finger-Stick Blood Microvolumes: Simple, Sensitive, and Suitable for Any Laboratory

**DOI:** 10.3390/antiox15040483

**Published:** 2026-04-14

**Authors:** Daniela Giustarini, Graziano Colombo, Isabella Dalle-Donne, Ranieri Rossi

**Affiliations:** 1 Department of Biotechnology, Chemistry and Pharmacy, University of Siena, 53100 Siena, Italy; 2 Center for Colloid and Surface Science (CSGI), University of Florence, 50019 Florence, Italy; 3 Department of Biosciences, University of Milan, 20133 Milan, Italy

**Keywords:** reduced glutathione, glutathione disulfide, blood, biomarkers, oxidative stress, finger-stick

## Abstract

Blood glutathione (GSH), its oxidized form glutathione disulfide (GSSG), and especially the ratio of reduced to oxidized glutathione (GSH/GSSG) are recognized as robust biomarkers of oxidative stress. However, the broader application of these biomarkers has been limited by two major challenges: (1) the high risk of artifact formation during sample handling, which can artificially increase GSSG levels and bias redox balance measurements, and (2) the reliance on complex, instrument-intensive analytical procedures and the requirement for venous blood. We present an adaptation of the highly sensitive and easy-to-perform Tietze recycling method for microvolumes of blood. The challenge is to achieve accurate and precise measurements while avoiding artifacts, taking advantage of the high sensitivity of this enzymatic recycling analytical procedure. The method uses a simplified sample preparation protocol compatible with small blood volumes (up to 10 μL) and requires only basic laboratory equipment, such as a standard spectrophotometer or microplate reader. As this is an enzyme-based assay, we also carefully evaluate the main factors that can affect the measurements. This novel procedure provides a practical tool for monitoring GSH/GSSG as a biomarker of oxidative stress in various experimental settings by eliminating the need for trained personnel for blood sampling (it is suitable for capillary blood), minimizing discomfort for subjects, and avoiding complex procedures or instruments for analyte detection.

## 1. Introduction

Glutathione is a tripeptide composed of glutamate, cysteine, and glycine and is one of the most important endogenous antioxidants in cells and biological fluids. It plays a central role in protecting cells from oxidative damage by neutralizing reactive oxygen species (ROS) and maintaining cellular redox balance. Glutathione acts both directly as a free radical scavenger and indirectly as a cofactor for antioxidant enzymes such as glutathione peroxidase. During its antioxidant activity, reduced glutathione (GSH) is converted to its oxidized form, glutathione disulfide (GSSG), through the formation of a disulfide bond between two GSH molecules [1].

The accumulation of GSSG and the resulting decrease in the GSH/GSSG ratio serve as sensitive indicators of the cellular redox state. Circulating blood continuously interacts with peripheral tissues and can therefore integrate ROS and other redox-active metabolites released into the systemic circulation. Consequently, the blood GSH/GSSG ratio provides a systemic measure of redox homeostasis that may reflect the oxidative status of organs and tissues that are otherwise difficult to access directly. This parameter is frequently used in research to assess pathological conditions associated with redox imbalance, such as inflammation, aging, and neurodegenerative and cardiovascular diseases [2,3,4].

Although the blood GSH/GSSG ratio is considered an excellent biomarker of oxidative stress and is widely used, its clinical potential remains somewhat underexploited. This is due to the inherently high physiological ratio between GSH and GSSG (>500), which makes measurement extremely prone to artifacts. The main methodological problem with this measurement arises from the acidic deproteinization step, which is the most common procedure in the pre-analytical phase. Acid treatment of biological matrices has been shown to oxidize the –SH group, probably as a consequence of ROS release, thus artificially forming GSSG [5]. We have demonstrated the impact of this process in our previous publication, indicating that the oxidation of 2% GSH would result in about 500% bias in GSSG analysis [6], distorting the actual GSH/GSSG ratio. This has also been reported for matrices other than blood, although to a different extent [7]. Additionally, measurement of the GSH/GSSG ratio is influenced by the interval between blood collection and sample stabilization, primarily because intraerythrocytic glutathione reductase can significantly reduce GSSG back to GSH. This phenomenon was verified by sample pre-treatment with the glutathione reductase (GR) inhibitor carmustine (BCNU) [5]. We also demonstrated that both these methodological problems can be prevented by sample pre-treatment with *N*-ethylmaleimide (NEM), as it is highly membrane permeable, binds GSH very rapidly, and is highly efficient in inhibiting GR. It is important to emphasize that GSH conjugation must be performed immediately after blood collection; delayed conjugation, especially if performed after sample acidification, can cause a significant artificial increase in GSSG, making this parameter unreliable as a biomarker of oxidative stress [8].

Over time, numerous methods have been developed to measure GSH and GSSG in blood. These include spectrophotometric and fluorimetric assays, as well as high-performance liquid chromatography (HPLC) coupled with various detection techniques such as electrochemical, fluorimetric, and mass spectrometric detection [3,9,10,11,12,13]. In addition, many commercially available kits, based primarily on spectrophotometric or fluorimetric principles, are widely used for this purpose. One of the most widely used approaches is the enzymatic recycling assay developed by Tietze, which relies on the enzymatic reduction of GSSG by glutathione reductase. In this assay, GSH reacts with 5,5′-dithiobis-(2-nitrobenzoic acid) (DTNB) to form GSSG and 2-nitro-5-thiobenzoate (TNB), the latter exhibiting strong absorbance at 412 nm [14]. In this method, total glutathione (GSH + GSSG) is measured, with GSSG usually quantified separately after GSH has been masked to prevent its reaction with DTNB.

However, most existing analytical approaches are prone to artifacts (e.g., omission or delayed addition of NEM), require relatively large blood volumes (hundreds of microliters), and depend on expensive instrumentation and highly trained personnel. Among recently published papers, there are some notable exceptions, such as a study describing a method based on capillary electrophoresis to detect GSH and GSSG in blood [15]. In this case, the problem of artificial GSH oxidation in the pre-analytical phase is considered and addressed. Additionally, Ten-Domenech et al. describe a method for analyzing capillary blood after absorption on filter paper and subsequent HPLC-MS/MS measurement using stable isotope dilution, with NEM pre-adsorbed on the filter paper [16].

Here, we propose a new artifact-free protocol for use with microvolumes of blood. Specifically, we fully exploited the potential of the Tietze method, which is notable for its simplicity, as it is spectrophotometric (requiring no expensive equipment or specialized personnel), and for its high sensitivity, resulting from the cyclic nature of the reaction, where both GSH and GSSG continuously generate TNB. It is important to note that, as a recycling assay based on enzymatic activity, this approach can be a double-edged sword. While it offers excellent sensitivity, any additional substance present in the reaction mixture may interfere with the enzyme kinetics and, consequently, with the measured concentrations. For this reason, we carefully evaluated all potential sources of interference, such as traces of NEM (which strongly inhibits GR), solvents used for NEM extractions, the impact of temperature, and other experimental conditions that could compromise accuracy and precision. The development of reliable new methods suitable for microvolumes of blood represents a significant advance, as it expands the field of application to a wide range of clinical and experimental studies.

## 2. Materials and Methods

### 2.1. Materials and Equipment

All reagents were purchased from Merck Italy (Milan, Italy). HPLC analyses were performed using an Agilent 1200 instrument equipped with UV-Vis and fluorometric detectors (Agilent Technologies, Milan, Italy). Chromatographic separations were carried out on a Zorbax Eclipse XDB-C18 4.6 × 150 mm, 5 μm column (Agilent Technologies, Milan, Italy). Spectrophotometric analyses were conducted on a UV-Vis spectrophotometer Jasco, V-750 (Jasco Europe, Cremella (LC), Italy). Blood samples in K_3_EDTA were obtained from the local blood bank.

### 2.2. Principle of the Enzymatic Recycling Assay for Total GSH and GSSG

The analytical method was based on a well-established enzymatic recycling reaction [14]. Briefly, GSH is oxidized to GSSG while reducing DTNB to TNB, a chromogenic product with an absorbance peak at 412 nm. GSSG is then reduced back to GSH by GR in the presence of NADPH, maintaining a continuous cycle. Absorbance was measured kinetically at 412 nm for 120 s, and the concentrations of total GSH and GSSG were calculated using a calibration curve. All reactions were performed at 25 °C.

### 2.3. Influence of Different Acids

1.5% (*w*/*v*) solutions of trichloroacetic acid (TCA), perchloric acid (PCA), metaphosphoric acid (MPA), and sulfosalicylic acid (SSA), adjusted to pH 7.4 with 2 M Tris and containing either 0.2 μM or 0.1 μM GSSG, were added to the cuvette containing 350 μL of 0.2 M Na^+^/K^+^ phosphate buffer, pH 7.4, and measured as described below.

### 2.4. Inhibition Caused by the Solvents or the Presence of N-Ethylmaleimide

Solutions of 1.5% (*w*/*v*) TCA adjusted to pH 7.4 with 2 M Tris and containing 0.25 mM GSSG were measured as described for GSSG blood analyses after NEM extraction with five volumes of different organic solvents (octanol, ethyl acetate, chloroform, and dichloromethane). Tubes were shaken for one minute, then centrifuged for 10 s at 10,000× *g* to separate the two phases, and the solvent was carefully aspirated and discarded. The amount of NEM remaining after each extraction was evaluated by measuring its concentration in the aqueous phase with HPLC as previously described [17].

### 2.5. Evaluation of Plasma Disulfide Influence

The influence of plasma disulfides was assessed by adding cystine (CySS), homocystine (HcySS), and cystinylglycine (CySSGly) to the cuvette containing all reagents used for GSSG blank reaction detection (see below). The final concentrations in the cuvette were based on physiological levels in humans (approximately 50 μM CySS, 2 μM HcySS, and 3 μM CySSGly [18]) and were increased up to fiftyfold. For comparison, the kinetics of reactions containing all reagents for blank reaction analysis (see below) were also measured.

### 2.6. K_M_ Determination for GSSG in the Glutathione Reductase Stock Solution, Temperature Effects, and Calibration Curves

The enzymatic activity of the GR batch used in this study was measured by spectrophotometric analysis of NADPH oxidation at a wavelength of 340 nm [19]. Activity was assessed at different temperatures by setting the thermostated spectrophotometer to the indicated values. To calculate the K_M_, GSSG was added to the cuvette at various concentrations (5, 10, 20, 35, 70, 150, and 500 μM), while NADPH was maintained at a constant concentration of 250 μM. The reaction was monitored for 30 s, and the kinetics were used to calculate activity.

Calibration curves were prepared with serial dilutions of GSH (from 2 to 0.1 μM) and GSSG (from 1 to 0.03 μM) in water and 1.5% (*w*/*v*) TCA, respectively. The values obtained from ΔAbs/min were used to evaluate linearity.

### 2.7. Evaluation of the Kinetics of GSH Alkylation by NEM

Blood samples (collected in evacuated plastic tubes with K_3_EDTA) were obtained from donors scheduled for blood donation. Blood hemolysates (20 μL + 200 µL of 10 mM Na^+^/K^+^ phosphate buffer, pH 7.4) were treated with 0.25, 0.5, 1, 2, 5, or 10 mM NEM (final concentrations). After 0.5, 1, 2, 5, 10, or 30 min of incubation, the samples were acidified by adding 7 µL of 60% (*w*/*v*) TCA, shaken for 30 s, and centrifuged at 10,000× *g* to remove proteins. GSH traces in the clear supernatant were measured by HPLC with fluorescence detection as previously described [20].

### 2.8. Blood Collection and Stabilization for GSH and GSSG Analysis

Twenty microliters of collected blood were immediately diluted in 200 µL of 10 mM Na^+^/K^+^ phosphate buffer, pH 7.4. This solution was then divided into two aliquots: 0.15 mL was used for GSSG analysis, while the remainder was used to measure total GSH and hemoglobin (Hb) concentration and stored at −20 °C until analysis. Samples for GSSG detection were immediately spiked with 2 µL of 200 mM NEM, and the mixture was gently shaken for 120 s to derivatize free thiols. The samples were acidified by adding 5 µL of 60% (*w*/*v*) TCA, shaken for 30 s, and centrifuged at 10,000× *g* to remove proteins. The supernatant was stored at −20 °C until analysis.

### 2.9. GSH Analysis and Hemoglobin Concentration

For GSH analysis, 2 μL of hemolysate was added to a cuvette containing 500 μL of 0.2 M Na^+^/K^+^ phosphate buffer, pH 7.4. Total GSH was then measured by adding 0.15 mM DTNB and 0.15 mM NADPH (final concentrations, from 20 mM stock solutions). The reaction was initiated by adding GR at a final concentration of 0.2 U/mL (from a 10 U/mL stock solution) and monitored for 2 min at 412 nm. The slopes were used for quantification after subtraction of the value obtained in a blank analysis (without the blood sample). GSH was determined by subtracting the GSSG concentration measured in the same sample [8].

Hemoglobin concentration was determined spectrophotometrically in the wavelength range of 500–700 nm after conversion to methemoglobin by the cyanide method [21] using 40 μL hemolysate.

### 2.10. GSSG Analysis

To measure GSSG, 750 μL of octanol was added to the tube containing the acidified supernatant, and the mixture was shaken vigorously for 2 min. The sample was then centrifuged for 30 s to ensure proper phase separation, and the supernatant was carefully removed. This extraction step was repeated three times. Finally, 150 μL of the sample was added to a cuvette containing 350 μL of 0.2 M Na^+^/K^+^ phosphate buffer, pH 7.4, and 5 μL of 2 M Tris base to neutralize the acidity of the sample. DTNB, NADPH, and GR were then added, and the reaction was monitored as described above for GSH.

### 2.11. Validation of the Method

Precision and accuracy were evaluated using 1 mL of human blood (from a blood bank) hemolyzed by adding 2 volumes of 10 mM Na^+^/K^+^ phosphate buffer, pH 7.4. The hemolysate was centrifuged at 20,000× *g* for 20 min to remove membranes and debris and then cleared of low-molecular-mass thiols and disulfides by gel filtration (PD 10 desalting columns) using 50 mM Na^+^/K^+^ phosphate buffer, pH 7.4, as the eluent. The eluted samples were adjusted to a hemoglobin content of 15 mg/mL (corresponding to a final dilution of 1:10) by adding H_2_O. GSH and GSSG were added to 0.2 mL hemolysate aliquots at various final concentrations, and the analytes were measured after TCA precipitation and NEM extraction with octanol according to the proposed protocol. Specifically, GSH was added at final concentrations of 50, 125, 250, and 500 µM, and GSSG at 0.1, 0.3, 0.75, and 2 µM. Precision is expressed as the relative standard deviation (RSD). Accuracy is expressed as relative error (RE) and calculated using the following formula: [(mean observed concentration − spiked concentration)/(spiked concentration) × 100%].

The lowest limit of quantification (LLOQ) was defined as the lowest concentration of added GSH or GSSG that resulted in kinetics at least four times greater than the blank reaction.

### 2.12. Comparison with the Reference Method for Blood GSH and GSSG

GSH and GSSG quantification was performed on the same blood samples using both the newly developed method and the reference HPLC method previously validated by our group [8]. For the HPLC reference procedure, 0.3 mL of whole blood was treated with 100 μL of 310 mM NEM and, after 30 s of vigorous shaking, acidified with TCA. The GS-NEM derivative was measured in the supernatant by UV-Vis HPLC at a wavelength of 265 nm. GSSG was quantified after reduction to GSH by dithiothreitol and subsequent labeling with monobromobimane, using a fluorescence detector [20]. Chromatographic separations were carried out on a C18 column. Agreement between the GSH and GSSG levels measured by the two procedures was evaluated using Bland–Altman analysis [22].

### 2.13. Treatment with Tert-Butyl Hydroperoxide

Three milliliters of blood were placed in a tube connected to a precision syringe that delivered a solution of *tert*-butyl hydroperoxide (*t*-BOOH) diluted in physiological saline to a final concentration of 2 mM. The infusion rate was 1 µL/min. The tube was kept under gentle rotary agitation (200 rpm) in a chamber at 37 °C. At specified times, GSH and GSSG levels were measured using the described method.

### 2.14. Statistics

Linear regression analyses were performed using the general equation y = ax + b, where a is the slope and b is the intercept with the *y*-axis. Data are expressed as mean ± SD. A value of *p* < 0.05 was considered statistically significant.

## 3. Results and Discussion

**Figure 1:**
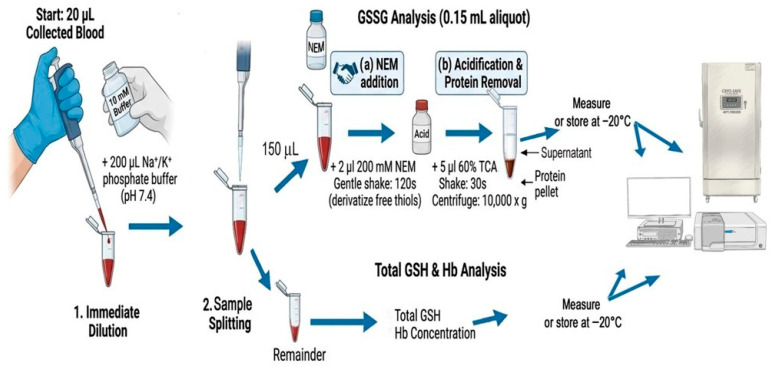
**Flow diagram showing the sequential** **processes of the protocol.**

The proposed protocol is designed to measure GSH and GSSG in microvolumes of whole blood. As shown in the flow diagram (Figure 1), the blood is first diluted 1:10 in a hypotonic solution (typically 20 μL of blood in 0.2 mL of solution). The resulting hemolysate is then divided into two aliquots: one aliquot of 0.15 mL is immediately treated with NEM for thiol blocking and deproteinized with TCA after 2 min; this fraction is used for GSSG analysis. The remaining sample is reserved for GSH and Hb quantification. Analyses can be performed on fresh samples or after storage; stability data confirm that samples stored at −20 °C remain suitable for analysis for at least 6 months (Figure S1). All measurements are performed spectrophotometrically using a modified version of the classic Tietze method [14], optimized for microvolume applications. Because the method couples DTNB conjugation with GSSG reduction, it measures total GSH (tGSH, i.e., GSH + GSSG). Therefore, blood samples are split into two aliquots, and GSSG is measured in the second sample. GSH is then calculated as the difference between tGSH and GSSG.

This recycling method offers a significant advantage in sensitivity; however, its main disadvantage is that, because it relies on an enzyme-catalyzed reaction, any substance present in the cuvette, as well as temperature and pre-analytical procedures, can strongly influence the results. In particular, these variables can affect GSSG measurement, since in our protocol the sample amount required for analysis constitutes approximately one-third of the reaction volume in the cuvette. The initial microvolume of blood was diluted to facilitate accurate pipetting and to enable liquid-phase extraction of NEM. Consequently, to achieve adequate GSSG concentrations in the reaction mixture, the sample volume added to the cuvette was increased. Under these conditions, both the acids used for protein precipitation and the solvents used for NEM extraction could affect assay performance.

### 3.1. Pre-Analytical Variables

Protein precipitation by acid treatment is an almost unavoidable step in the procedure for GSSG detection. Although ultrafiltration could in principle be used, we rejected this option because it is both costly and time-consuming. Since the acid-precipitated sample ultimately constitutes a significant fraction of the cuvette mixture, it is important to evaluate which acid interferes least with glutathione reductase activity. Table 1 presents the results of the experiment comparing the most commonly used acids with a buffered control solution. It is evident that all acids inhibit the reaction, even though all samples were adjusted to pH 7.4 before being added to the cuvette. This is probably due to the known chaotropic effect exerted by several acids, meaning they disrupt the hydrogen-bonding network of the water surrounding the enzyme. This interferes with the precise binding of the substrates (GSSG and NADPH) within the catalytic pocket of GR. Even if the bulk pH was maintained in the optimal range (pH 7.5), residual acid anions can create localized ionic environments that alter the K_M_ or Vmax of the enzyme. However, trichloroacetic acid produced a markedly lower degree of inhibition compared to the others, effectively precipitated blood proteins, and was selected for deproteinization.

NEM pre-treatment of the sample is required to prevent nonspecific GSH oxidation to GSSG during the pre-analytical step [8]. It is essential to remove excess NEM, as it is a potent inhibitor of glutathione reductase. Solid-phase extraction cannot be used because NEM has a partition coefficient (Kow) close to one; therefore, we evaluated the efficiency of the simpler and more cost-effective liquid–liquid extraction. Surprisingly, dichloromethane (commonly used for NEM extraction) inhibited more than 50% of GR activity (Table 2). Other solvents, such as chloroform, ethyl acetate, and ether, inhibited 60–85%. The most suitable solvent was octanol, which showed less than 15% inhibition, likely due to its lower miscibility with water (solubility in water is about 0.46 g/L). Additionally, it is a larger, long-chain molecule that is less likely to penetrate deeply into the narrow catalytic clefts of the enzyme compared to small, highly mobile molecules such as dichloromethane. We further optimized the number of extraction cycles by measuring the residual NEM after each step. As expected from the partition coefficient of NEM (Kow ≈ 1), a 1:5 extraction reduced NEM by approximately 80%. Therefore, three cycles were sufficient to decrease NEM to negligible levels. Since octanol does not significantly inhibit the enzyme, further purification steps such as evaporation or washing were omitted to minimize processing time.

### 3.2. Influence of Blood Disulfides and Temperature on Spectrophotometric Analysis

**Figure 2:**
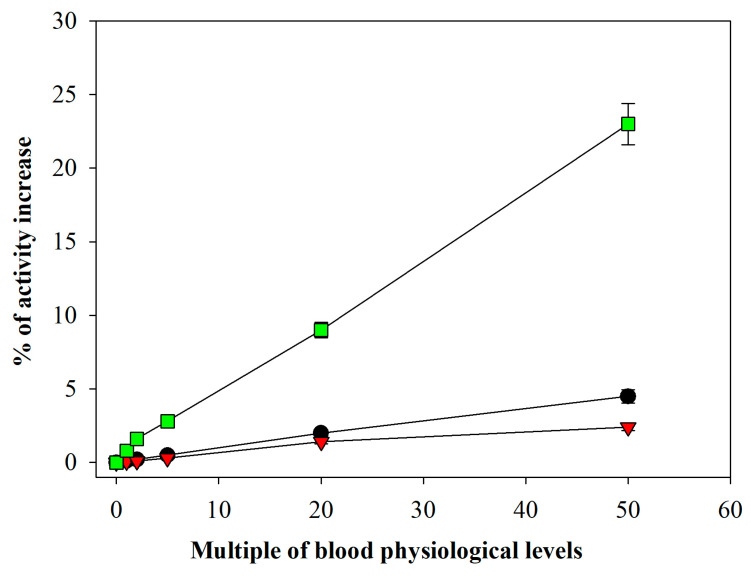
**Evaluation of the influence of plasma disulfides.** The blank reaction was supplemented with 1–50 times the physiological levels of cystine (black circles), cystinylglycine (green squares), and homocystine (red triangles), respectively. The final concentrations in the cuvette were 0.6, 1.2, 3, 12, and 30 μM for cystine; 0.025, 0.05, 0.12, 0.5, and 1.2 μM for homocystine; and 0.035, 0.07, 0.18, 0.70, and 1.8 μM for cystinylglycine. The contribution of multiples of the basal levels of low-molecular-weight plasma thiols in the reaction after the various dilutions is shown in the figure. Results are expressed as the percentage of ΔAbs/min increase with respect to the blank. Number of replicates = 4.

Because the method relies on detecting trace amounts of GSSG in blood, we investigated potential interference from other disulfides present at physiological concentrations in the assay. While the cellular fraction of blood contains minimal low-molecular-mass disulfides (LMM-SS) other than GSSG [23], significant levels are present in the extracellular compartment (for example, cystine is 20–60 μM). In fact, cystine, homocystine, and cystinylglycine occur at concentrations 2–25 times higher than GSSG in human blood [18]. Because the protocol depends on the reaction specificity of glutathione reductase toward GSSG, we sought to verify this property. Figure 2 shows the results of an experiment in which increasing concentrations of these disulfides were added to the reaction mixture in the cuvette. Glutathione reductase exhibited only minimal NADPH-mediated reduction of all tested disulfides, and only supraphysiological levels of cystinylglycine showed measurable reduction under these conditions. Therefore, this type of interference can be considered negligible.

The issue of temperature control must not be overlooked, as it greatly influences enzyme activity and, consequently, the accuracy of the method. In our study, we used a thermostated spectrophotometer; however, such equipment may not be available in all laboratories. The enzyme used is purified from baker’s yeast, and its maximal activity is expected at approximately 28–30 °C. Table 3 reports GR activity as a function of temperature. The data suggest that for accurate measurements, it is crucial to perform calibration curves at the same temperature, since, within the 18–30 °C range, the reaction rate increases 3–8% for each additional degree. Thus, thermostated devices are recommended, or, alternatively, reactions should be performed close to room temperature (20–22 °C); in this case, both calibration curves and sample measurements must be carried out at the same temperature. It should be emphasized that the buffer used for analysis must be maintained at the same temperature as the reaction.

### 3.3. K_M_ for Glutathione Reductase and Calibration Curves

**Figure 3:**
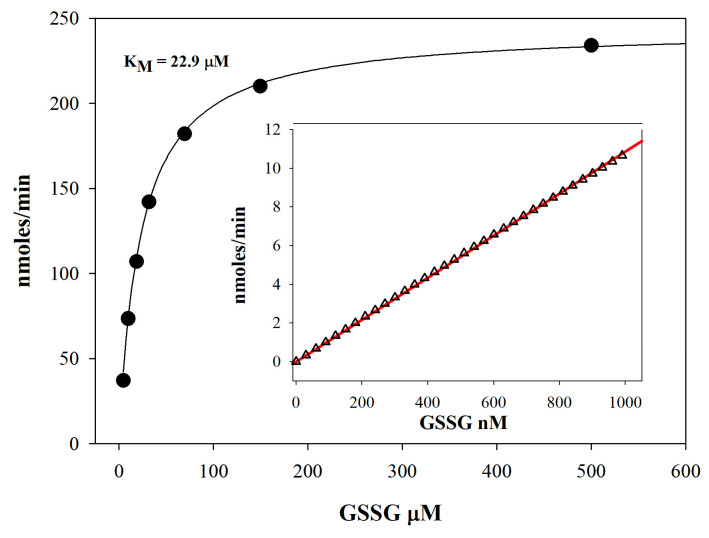
**Michaelis–Menten kinetics of glutathione reductase.** Reaction rates were measured at final GSSG concentrations ranging from 0 to 500 μM. Circles represent experimental data points, and the solid line shows the Michaelis–Menten fit. The inset displays an expanded view of the 0–1000 nM range: triangles indicate the Michaelis–Menten fit within 1–1000 nM, and the straight line represents the linear regression.

Due to the enzymatic nature of the method, the relationship between substrate concentration and reaction rate is non-linear and follows Michaelis–Menten kinetics. Therefore, it is essential to determine the K_M_ value for GSSG for each specific batch of GR used in the assay under the exact experimental conditions. Only if the K_M_ value is significantly higher than the measured glutathione concentration range will the initial reaction rate approach linearity. However, if the K_M_ value is close to the measured analyte concentrations, this does not affect the method’s applicability but complicates the calibration curves, making them non-linear. Linearity of the slope is a prerequisite to facilitate the assay for GSH and GSSG analysis. Under these conditions, the method can provide accurate and precise quantification and minimize possible deviations due to saturation kinetics. As shown in Figure 3, the enzyme exhibits a K_M_ of 22.9 µM for GSSG. This value confirms the enzyme’s kinetic behavior in our experimental setup and supports the assumption of nearly linear reaction rates at substrate concentrations well below this K_M_ (Figure 3, inset).

**Figure 4:**
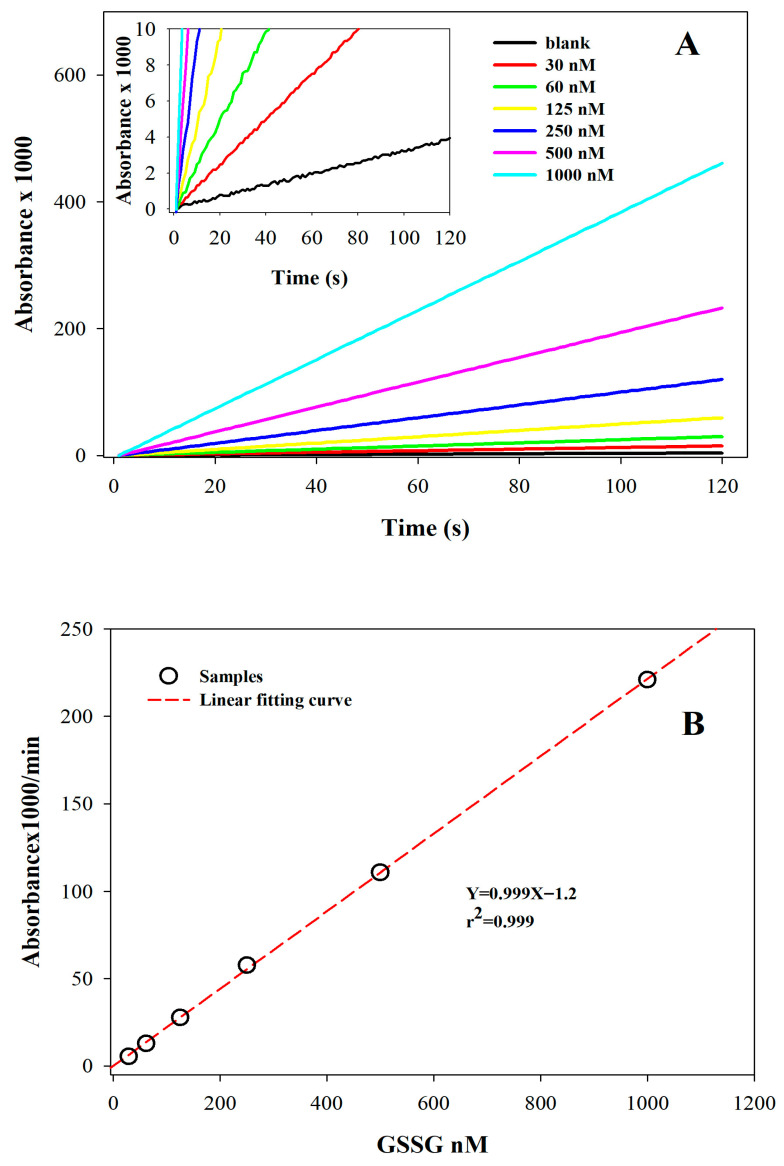
**Calibration curves for GSSG and assessment of linearity:** (**A**) GSSG concentrations were determined in standard samples prepared in the 0–1 μM range using the GSH recycling method. The inset shows an enlarged view of the data (0–10 absorbance units × 1000). (**B**) Linear regression (dotted red line) fits the ΔAbs × 1000/min values for each GSSG standard (open circles). The data used for the regression were obtained from the analyses shown in panel (**A**).

The spectrophotometric curves obtained by applying the assay to GSSG standard solutions (Figure 4A,B) showed a linear increase in absorbance over time across the concentration range of 30 to 1000 nM (r^2^ > 0.999). Similarly, a linear increase was observed for GSH in the 0.1–2 µM concentration range (r^2^ > 0.999). These values correspond to a concentration range of 0.25–5 mM for GSH and 1–30 µM for GSSG in whole blood, thus covering the full spectrum of physiological and pathological concentrations expected in human blood samples [15,16,24].

A slow, spontaneous increase in absorbance was observed due to the activity of glutathione reductase on DTNB in the absence of substrate; this was considered a blank reaction (inset in Figure 4A). Because both the blank reaction and the sample kinetics are highly sensitive to the specific concentrations of GR and DTNB, we optimized these parameters to achieve the highest analytical sensitivity for glutathione while keeping the blank reaction at negligible levels. Specifically, this background signal was consistently at least three times lower than the response measured at the lowest concentration tested.

### 3.4. Kinetics of GSH Alkylation by NEM

**Figure 5:**
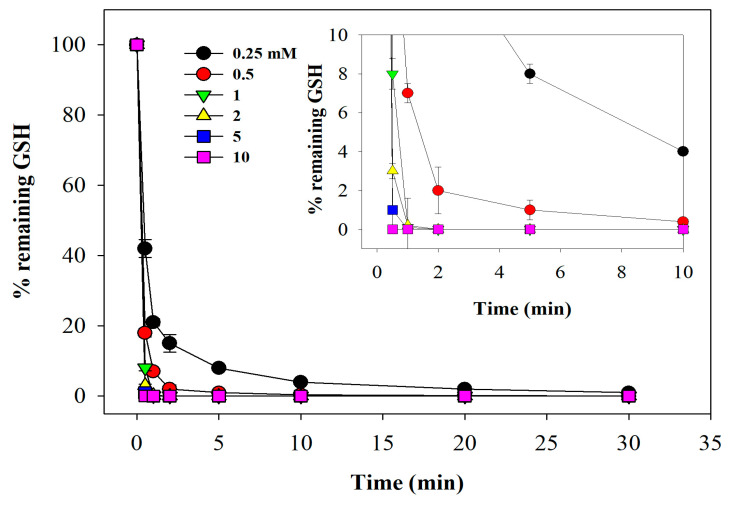
**Kinetics of GSH alkylation by NEM.** Various final concentrations of NEM (0.25–10 mM) were added to 1:10 hemolysates and incubated for 0.5–30 min. The remaining GSH was then quantified by HPLC. Number of replicates = 5.

NEM is considered essential for accurate GSSG analysis and offers several advantages over other alkylating agents, including faster reaction rates and improved membrane permeability [25]. Because it is used to eliminate all GSH for accurate GSSG quantification, one might assume that using a large excess and allowing it to react for an extended period would be sufficient. However, this approach has two drawbacks: (i) high concentrations are more difficult to remove, and residual NEM may inhibit GR; (ii) NEM also reacts, though more slowly, with the amino group of the two glycine residues in GSSG [26], forming a stable *N*-alkylated succinimide adduct that is not a substrate for GR. Therefore, we sought optimal conditions for sample pre-treatment with NEM for GSSG determination to ensure complete alkylation of GSH, making it unavailable for the reaction. To achieve this, we conducted an experiment in which samples were treated with increasing concentrations of NEM and incubated for various times (Figure 5).

GSH disappeared rapidly, and incubation with 1 mM NEM for 60 s at room temperature eliminated all GSH. Given that the mean concentrations of GSH and protein thiols (primarily hemoglobin and albumin) in human blood are approximately 1 mM and 5 mM, respectively [27,28], the expected final values in our reaction mixture after sample dilution are 0.1 mM and 0.5 mM. Since GSH reacts significantly faster with NEM than protein-bound thiols, a 1 mM NEM concentration provides a substantial stoichiometric excess (10-fold relative to GSH), ensuring rapid and complete derivatization. To ensure complete alkylation, we used 2 mM NEM for 2 min.

### 3.5. Method Validation

The precision and accuracy of the method were evaluated using hemolysates depleted of physiological GSH and GSSG, spiked with four known concentrations of these analytes (Table 4). Intra- and inter-day relative standard deviations (RSDs) were less than 5% for all samples tested, indicating low within-sample variability and a high degree of analytical precision. The measured values matched the expected concentrations, demonstrating high accuracy for both GSH and GSSG determinations. This experimental setup also allowed determination of the LLOQ for the method, which was 75 µM for GSH and 0.5 µM for GSSG in whole blood. These values are well below the physiological concentrations typically found in healthy individuals and confirm that the method is suitable for accurate and sensitive quantification under both physiological and pathological conditions. The recovery of GSH and GSSG was close to 100% for all concentrations tested (Table 4).

### 3.6. Comparison Between the New Protocol and the Reference Method

**Figure 6:**
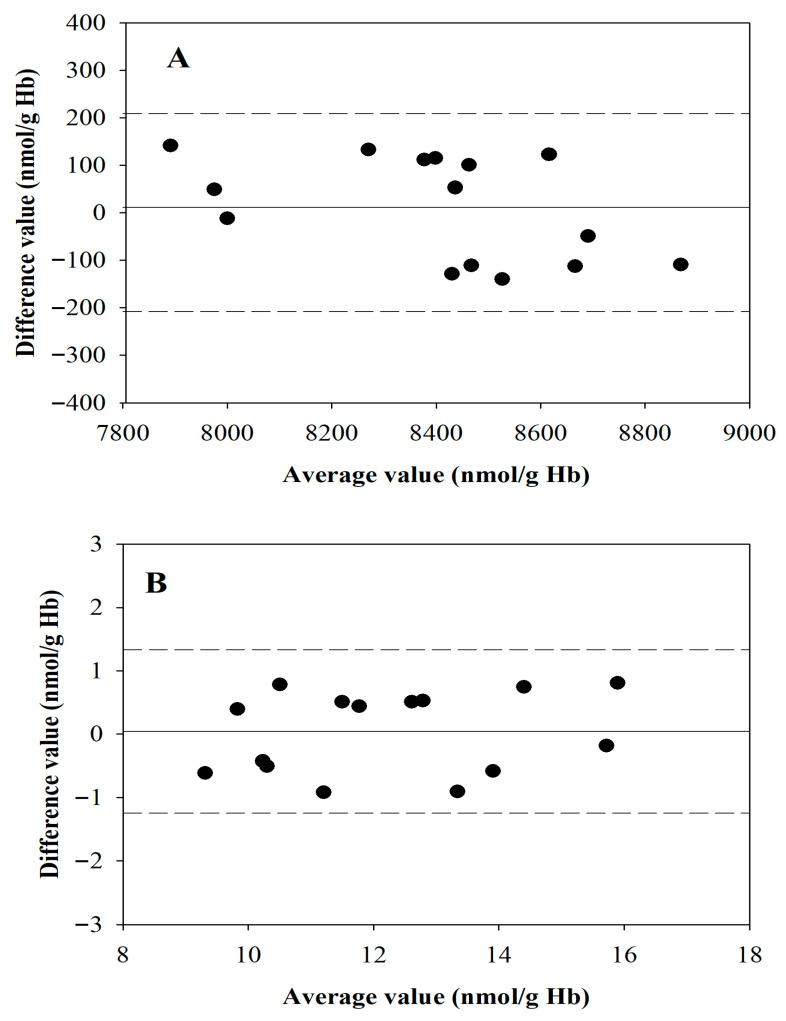
**Bland–Altman plot for GSH and GSSG.** GSH (panel (**A**)) and GSSG (panel (**B**)) were measured in human blood using the present protocol and an HPLC reference method [8].

To further validate the proposed method, it was compared with an HPLC-based reference method. GSH and GSSG levels were measured in whole blood samples from 15 healthy volunteers. Each sample was analyzed using both a previously published HPLC method [8] and the present method, with the latter applied to only 20 µL of blood to simulate a finger-stick sample. The values showed a high correlation (r = 0.9746 for GSH and 0.9693 for GSSG). Bland–Altman analysis revealed a high degree of agreement between the two methods (Figure 6A,B).

The mean bias was 13.1 nmol/g Hb for GSH and 0.028 nmol/g Hb for GSSG. All data were within the limits of agreement (range: bias ± 223 for GSH and bias ± 1.31 for GSSG), confirming the accuracy of the enzymatic recycling assay for microvolume samples. The mean values measured with the present method were 8307 ± 292 nmol/g Hb for GSH and 12.2 ± 0.5 nmol/g Hb for GSSG, which agree with those published previously [8]. Considering that the reported LLOQ of the method for these two parameters is much lower than the reference concentrations, this reinforces the notion that it is well-positioned to capture both baseline values and the redox shifts typical of oxidative stress.

### 3.7. What Is the Lowest Blood Volume at Which This Method Can Be Applied Reliably?

**Figure 7:**
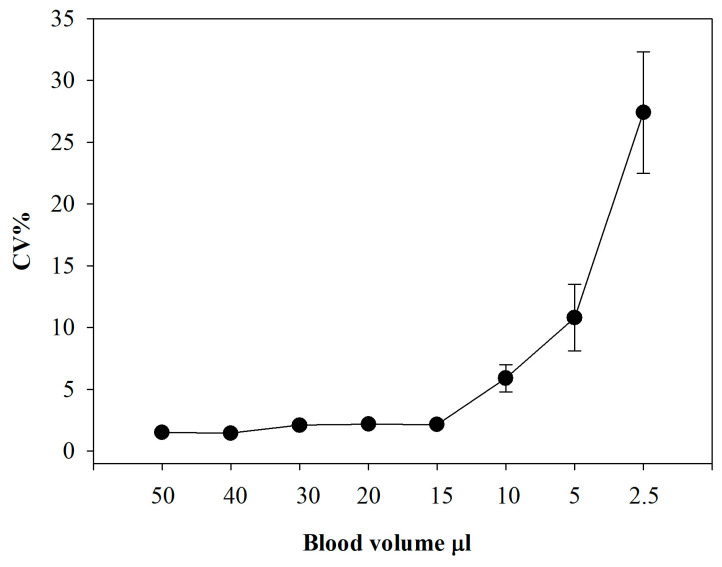
**Effect of sample volume on GSSG analysis.** GSSG was measured by the GSH recycling method using different blood volumes. Number of replicates = 5 for each volume. The coefficient of variation (CV%) is indicated for each analysis point.

Total GSH is measured by the recycling assay in blood hemolysates, so neither sample alkylation (and the associated NEM extraction steps) nor acidification is required. More importantly, GSH concentrations are at least two orders of magnitude higher than those of GSSG. Therefore, sub-microliter volumes of blood can be used for the assay. The challenge arises with GSSG, since the above-mentioned pretreatments are necessary. We therefore assessed how decreasing the volume of analyzed blood affects the precision of the method. Figure 7 shows the results of an experiment in which blood volumes ranging from 50 to 2.5 µL were tested.

The data indicate that the method maintains very good precision down to 15 µL (CV < 3%), remains acceptable at 10 µL (CV 6.5%), but becomes imprecise at 5 µL (CV 18%). These findings are consistent with our earlier observations, where at low cuvette concentrations of GSSG, the reaction kinetics began to approach those of the blank. It is important to emphasize that one of the major strengths of the method is its robustness with respect to sample volume. Although analyses are typically performed using defined amounts of whole blood (usually 20 µL), the method remains fully applicable even when the exact sample volume is unknown, as the results are normalized to hemoglobin concentration. The only limitation concerns the lower limit of quantification. Blood glutathione levels are usually normalized to either sample volume or hemoglobin concentration. We opted for the latter approach because red blood cells make up about 99% of the cellular fraction of blood, and GSH is present in the extracellular compartment at concentrations almost three orders of magnitude lower [29]. For samples with a significantly low hematocrit (e.g., ≤30%), we recommend slightly increasing the sample volume to 20–25 µL to maintain optimal precision and ensure a sufficient analyte load for detection. Conversely, the experimental conditions are suitable for high hematocrit samples (up to 65%).

### 3.8. NEM Treatment Prevents Both the Oxidation of GSH and the Reduction of GSSG, Which Are Methodological Artifacts

**Figure 8:**
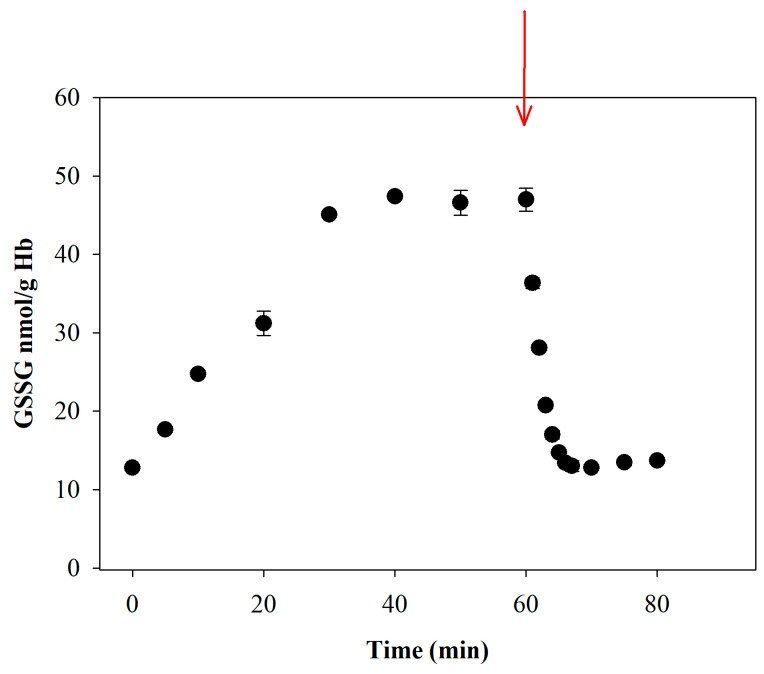
***tert*-Butyl hydroperoxide-induced oxidations and effect of delayed NEM treatment.** *Tert*-Butyl hydroperoxide (*t*-BOOH) was diluted in physiological saline to a final concentration of 2 mM and added to 1 mL of blood at an infusion rate of 1 µL/min for 60 min (the red ink indicates the stopping time for the treatment). At specified times, 20 μL of blood was collected, diluted, treated with NEM, and GSSG was measured. Number of replicates = 4.

It should be emphasized that for GSH/GSSG to serve as a robust and reliable biomarker of oxidative stress in blood, it is essential to prevent both artifactual oxidation of GSH and potential reduction of pre-existing GSSG during the pre-analytical step. Blood is an actively metabolizing tissue and a physiological sink for ROS generated by other tissues; once withdrawn, the external ROS influx ceases. Consequently, any GSSG formed in vivo is rapidly reduced back to GSH by glutathione reductase, as shown in Figure 8. In the experiment reported here, we simulated mild oxidative stress, similar to what may occur in vivo, by treating blood with a slow, continuous flux of a solution containing an organic peroxide (*t*-BOOH). An immediate, significant increase in GSSG was detected, while GSH levels remained unchanged. After approximately 30 min from the start of infusion, the system appeared to reach equilibrium. At 60 min, the flux was stopped by removing the pro-oxidant stimulus (*t*-BOOH), and the GSSG was rapidly (within a few minutes) reduced back to GSH. Therefore, immediate dilution and NEM treatment not only prevent artifactual oxidation during sample handling but also counteract the often overlooked phenomenon of enzymatic reduction of pre-existing disulfides.

### 3.9. Method Advantages, Possible Applications, and Study Limitations

Here, we propose a validated protocol for GSH and GSSG detection in microvolumes of blood (as little as 10 μL), addressing the critical challenge of preventing artifactual oxidation of GSH to ensure reliable redox state measurements. The procedure is suitable for minimal blood collection procedures such as the finger-stick method. The protocol described in this study offers several notable advantages: (i) it does not require trained personnel; (ii) detection is accomplished by straightforward spectrophotometric measurement; and (iii) it is inexpensive, easily automated, and compatible with plate readers. The transition from venous blood sampling to capillary blood analysis represents a significant advancement in procedural simplification. Using microvolumes of blood to measure GSH/GSSG, such as those obtained from a finger stick, does not require trained personnel for sample collection, is minimally invasive and less uncomfortable for the donor, and enables repeated sampling at short intervals, making it especially suitable for long-term monitoring and point-of-care applications. The spectrophotometric analysis is best performed by directly monitoring the reaction at a 412 nm wavelength [8]. However, for equipment lacking this capability, a reliable kinetic slope can be obtained by manually recording the absorbance every 30 s for a total of 2 min. The same approach is applicable to standard microplate readers. This interval provides a sufficient number of data points to ensure linearity and allows the calculation of the reaction rate with high precision.

While the assay could technically be applied to isolated RBCs, we believe that whole blood analysis remains the most reliable approach for redox assessment. Since over 98% of blood glutathione is contained in erythrocytes, the concentration measured in whole blood directly and accurately reflects the intra-erythrocytic pool. Furthermore, the required centrifugation and washing steps to isolate RBCs are time-consuming and significantly increase the risk of ex vivo GSH auto-oxidation. This could artificially alter the GSH/GSSG ratio, compromising its validity as a sensitive biomarker of systemic oxidative stress.

The method we used has exceptionally high sensitivity, as it is not based on conventional endpoint titration but on a continuous recycling reaction. Theoretically, it should also be applicable to plasma, where physiological concentrations of GSH are typically low (2–3 μM), and GSSG represents about 20–30% of GSH. However, the expected GSSG concentrations in plasma would be critically close to the LLOQ of our assay, potentially reducing the precision and reproducibility of the results in this specific matrix.

While this study establishes the analytical robustness of the assay, a systematic comparison between capillary and venous blood was not performed. We recognize that capillary blood collection may introduce additional variables related to the shear forces applied to the finger, which should be considered. However, because all glutathione measurements were normalized to hemoglobin levels, the impact of sampling-site-related volume fluctuations is expected to be negligible. This aspect, common to all finger-stick methodologies, remains an important area for future clinical investigation.

In any case, the fact that our procedure is validated for microvolumes of blood makes it ideal for studies requiring numerous or frequent blood draws. Additionally, it is useful for investigations using microvolumes of blood collected from laboratory animals, further expanding its potential applications.

## Figures and Tables

**Table 1:** **Influence of different acids on GSSG measurement.** Acid solutions were buffered to pH 7.4 with 2 M Tris and contained GSSG at two concentrations. The GSSG concentration refers to the sample before addition to the cuvette. Number of repeated experiments = 4.

GSSG ^1^ Standard	Acid	Relative Kinetic %
100 nM	None	100
100 nM	TCA	79.6 ± 3.3
100 nM	PCA	21.1 ± 1.9
100 nM	SSA	36.1 ± 0.8
100 nM	MPA	48.8 ± 0.2
200 nM	None	100
200 nM	TCA	86.6 ± 1.0
200 nM	PCA	25.2 ± 2.0
200 nM	SSA	32.9 ± 0.5
200 nM	MPA	47.0 ± 1.4

^1^ Abbreviations: GSSG = glutathione disulfide; MPA: metaphosphoric acid; PCA: perchloric acid; SSA: sulfosalicylic acid; TCA: trichloroacetic acid.

**Table 2:** **Influence of different organic solvents on GSSG measurement.** GSSG was prepared at two concentrations, treated with 2.5 mM NEM in 1.5% (*w*/*v*) TCA adjusted to pH 7.4, and excess NEM was extracted with various organic solvents. The GSSG concentration refers to the sample before addition to the cuvette. Number of repeated experiments = 4. Results are expressed as a percentage of the ΔAbs/min value obtained by substituting acid solutions with phosphate buffer at pH 7.4.

GSSG Standard	Extraction	Relative Kinetic %
100 nM	None	100
100 nM	Octanol	94.2 ± 4.1
100 nM	Ethyl acetate	15.0 ± 4.3
100 nM	Chloroform	43 ± 3.6
100 nM	Dichloromethane	56 ± 9
200 nM	None	100
200 nM	Octanol	96.6 ± 3.2
200 nM	Ethyl acetate	11.3 ± 2.0
200 nM	Chloroform	41.9 ± 1.5
200 nM	Dichloromethane	50.7 ± 5.4

**Table 3:** **Effect of temperature on glutathione reductase activity.** Glutathione reductase activity was measured by spectrophotometry according to the procedure of Carlberg et al. [19]. The mixture in the cuvette was incubated for 10 min at the specified temperatures before the enzyme was added. Number of replicates = 3.

Temperature °C	Activity %
32	84.7 ± 2.5
30	100
28	89.0 ± 2.2
26	83 ± 0.8
24	76 ± 1.0
22	67 ± 0.8
20	54 ± 0.7
18	38 ± 0.1

**Table 4:** **Procedure validation.** Recovery, precision, and accuracy were calculated by adding standard amounts of GSH to hemolyzed blood samples collected from healthy donors. Analytes were added to the eluate at the following final concentrations, indicated by numbers 1–4: 50, 125, 250, and 500 μM for GSH and 0.1, 0.3, 0.75, and 2 μM for GSSG.

Analyte Addition	1	2	3	4
**Recovery % GSH**	98.8 ± 0.6	98.0 ± 0.7	97.5 ± 1.3	99.1 ± 1.7
**Recovery % GSSG**	96.7 ± 0.5	97.9 ± 0.4	98.3 ± 0.6	98.5 ± 0.9
**Precision (RSD, %)**				
Intra-day GSH	3.23 ± 0.51	3.17 ± 0.68	3.58 ± 0.55	2.87 ± 0.34
Inter-day GSH	2.25 ± 0.55	3.06 ± 0.40	2.88 ± 0.31	3.16 ± 0.47
Intra-day GSSG	3.55 ± 0.61	2.68 ± 0.41	3.66 ± 0.50	3.42 ± 0.76
Inter-day GSSG	2.96 ± 0.50	2.85 ± 0.44	2.74 ± 0.63	3.35 ± 0.41
**Accuracy (RE, %)**				
Intra-day GSH	−1.96 ± 0.54	1.88 ± 0.67	−1.64 ± 0.45	1.36 ± 0.61
Inter-day GSH	1.41 ± 0.66	2.05 ± 0.84	−2.03 ± 0.67	−1.40 ± 0.55
Intra-day GSSG	−5.01 ± 2.63	2.64 ± 1.03	−1.36 ± 0.44	−2.22 ± 0.47
Inter-day GSSG	−4.78 ± 3.27	−2.87 ± 1.11	−1.65 ± 0.75	1.94 ± 0.60

## Data Availability

The data presented in this study are available upon request from the corresponding author; the only data used in this study were to generate mean values.

## Supplementary Materials

The following supporting information can be downloaded at: https://www.mdpi.com/article/10.3390/antiox15040483/s1, Figure S1: Stability of GSH and GSSG in samples stored at −20 °C.

## Abbreviations

The following abbreviations are used in this manuscript:

GSH	Reduced glutathione
GSSG	Glutathione disulfide
GR	Glutathione reductase
DTNB	5,5′-dithiobis-(2-nitrobenzoic acid)
TNB	2-nitro-5-thiobenzoate
NEM	*N*-ethylmaleimide
TCA	Trichloroacetic acid
PCA	Perchloric acid
MPA	Methaposphoric acid
SSA	Sulfosalicylic acid
CySS	Cystine
HcySS	Homocystine
CySSGly	Cystinylglicine
RSD	Relative standard deviation
RE	Relative error
*t*-BOOH	*Tert*-butyl hydroperoxide
