# Supplementary material for: An Artifact-Free Assay for the GSH/GSSG Ratio Adapted for Finger-Stick Blood Microvolumes: Simple, Sensitive, and Suitable for Any Laboratory
Source: Antioxidants (Basel). 2026 Apr 14;15(4):483. doi: 10.3390/antiox15040483 (PMC13113195; doi:10.3390/antiox15040483)
Supplement: Supplementary file 1 [file antioxidants-15-00483-s001.zip › antioxidants-4194063-supplementary.pdf]

Article

An Artifact-Free Assay for the GSH/GSSG Ratio Adapted for Finger-Stick Blood Microvolumes: Simple, Sensitive, and Suitable for Any Laboratory

Daniela Giustarini[1]*, Graziano Colombo[2], Isabella Dalle-Donne[3] and Ranieri Rossi[4]

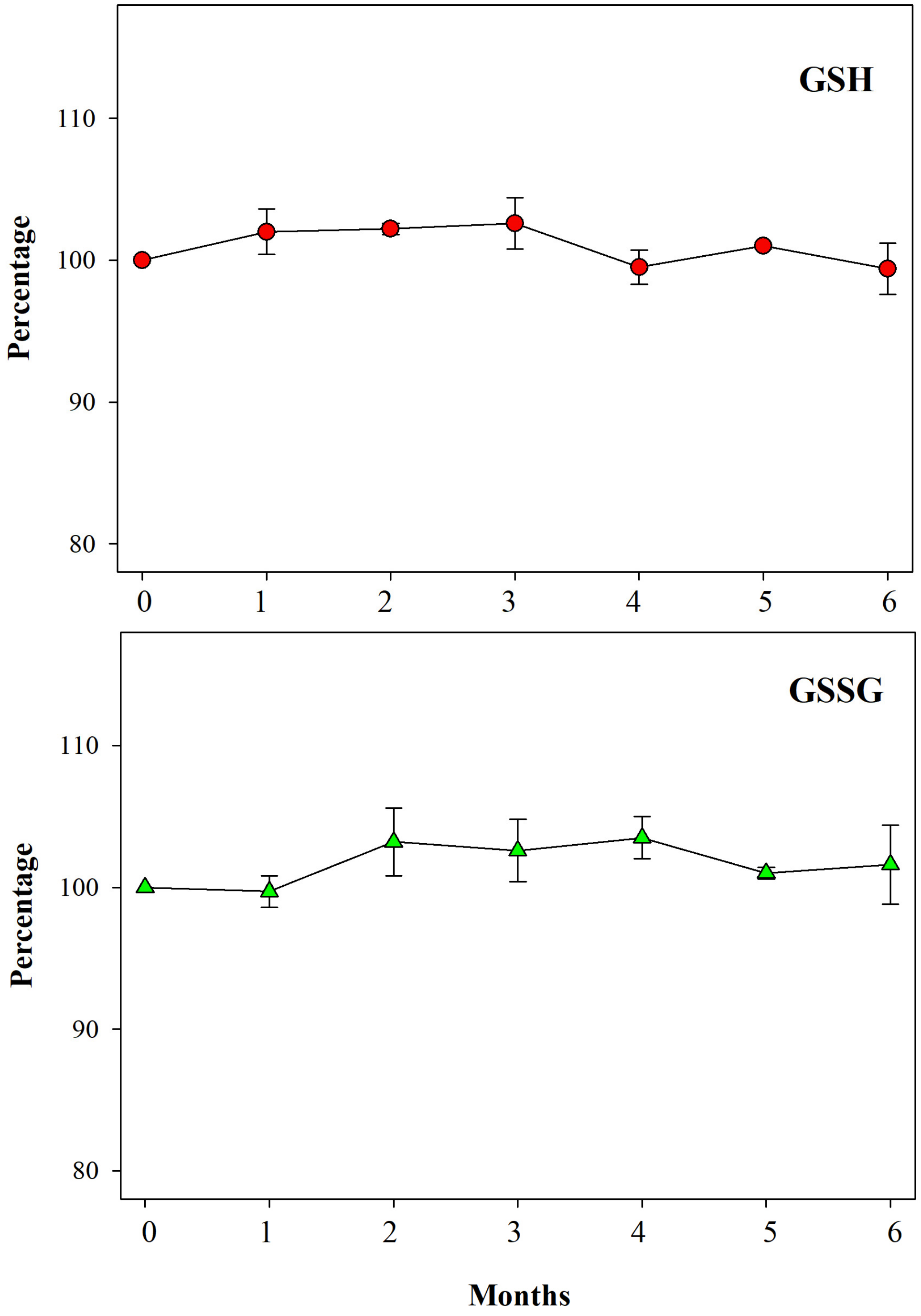

Figure S1. Stability of GSH and GSSG in samples stored at -20°C. Whole blood (0.2 ml) from four donors was hemolyzed by adding 2 ml of 10 mM Na^+/K^+ phosphate buffer (pH 7.4). Baseline GSH and GSSG levels were measured immediately after hemolysis. The remaining hemolysate was stored at -20°C. Frozen samples were thawed monthly and then refrozen to re-evaluate GSH and GSSG concentrations. Data represent the mean of four replicates.
